# An Unusual Progression of Bazex Syndrome (Acrokeratosis Paraneoplastica)

**DOI:** 10.7759/cureus.106783

**Published:** 2026-04-10

**Authors:** Benjamin Kahn, Blake C Van Noord, Sophia Skedros, Dionne Louis, Marcus B Goodman

**Affiliations:** 1 Dermatology, Goodman Dermatology, Roswell, USA; 2 Dermatology, Philadelphia College of Osteopathic Medicine, Philadelphia, USA

**Keywords:** acrokeratosis paraneoplastica, bazex syndrome, dermatopathology, oral cavity squamous cell carcinoma, paraneoplastic syndromes

## Abstract

Acrokeratosis paraneoplastica, also known as Bazex syndrome, is a rare paraneoplastic psoriasiform dermatosis that is strongly associated with squamous cell carcinoma (SCC) of mainly the head and neck. The disease typically progresses through three stages, initially affecting the fingernails, nose, and ears before spreading to the palms, soles, and eventually the trunk. We present an unusual progression of a psoriasiform rash in which the dorsal hands, trunk, and legs were primarily affected before involving the palmar hands and ears in a patient later diagnosed with oral SCC. This case also highlights the importance of maintaining a high index of suspicion for paraneoplastic conditions that could lead to more prompt diagnoses of internal malignancies and improve patient outcomes.

## Introduction

Paraneoplastic dermatoses are a heterogeneous group of cutaneous conditions that arise in association with internal malignancies but are not direct extensions, precursors, or metastases of the underlying tumor [1,2]. Their recognition is of considerable clinical importance, as cutaneous manifestations may precede the diagnosis of an occult malignancy by months to years, offering a critical window for early cancer detection and intervention [2,3]. Among the papulosquamous paraneoplastic dermatoses, acrokeratosis paraneoplastica-also known as Bazex syndrome-is a rare paraneoplastic condition most strongly associated with squamous cell carcinoma (SCC) of the upper aerodigestive tract [4,5].

The condition was first described in 1922 by Gougerot and Grupper, who reported an acral eruption in a patient with an internal malignancy [6]. In the 1960s, André Bazex formally characterized the association between the distinctive psoriasiform dermatosis and underlying neoplasia [7]. Despite its rarity, Bazex syndrome is the third most common paraneoplastic phenomenon associated with keratinocyte skin cancer [8]. It primarily manifests as erythematous to violaceous, psoriasiform plaques affecting acral regions-most characteristically the hands, feet, ears, and nose-and often involves nail dystrophy [5,9]. The condition chiefly affects Caucasian men, with a mean age of approximately 60 years, and alcohol or tobacco use disorder is a common comorbidity [4,9].

Bazex syndrome is classified as an obligate paraneoplastic dermatosis, meaning it occurs exclusively in the setting of an underlying malignancy [7]. Bolognia et al. found that cutaneous lesions preceded the diagnosis of the associated tumor in 63% of cases by an average of 11 months, and in 91% of patients, the eruption either improved with successful cancer treatment or persisted in the setting of refractory disease [5]. These findings underscore the potential of Bazex syndrome to serve as an early clinical marker of occult malignancy. However, because of the psoriasiform morphology, Bazex syndrome is frequently misdiagnosed as psoriasis, leading to delays in cancer detection [4,9]. Histopathology is non-specific and varied, further compounding the diagnostic challenge [9].

Given its rarity and the diagnostic pitfalls it presents, Bazex syndrome remains an underrecognized clinical entity [4,5]. We present a case of an unusual variant on the typical progression of Bazex syndrome and emphasize the importance of early recognition in facilitating prompt oncologic evaluation and management, ultimately improving patient outcomes.

## Case presentation

**Figure 1:**
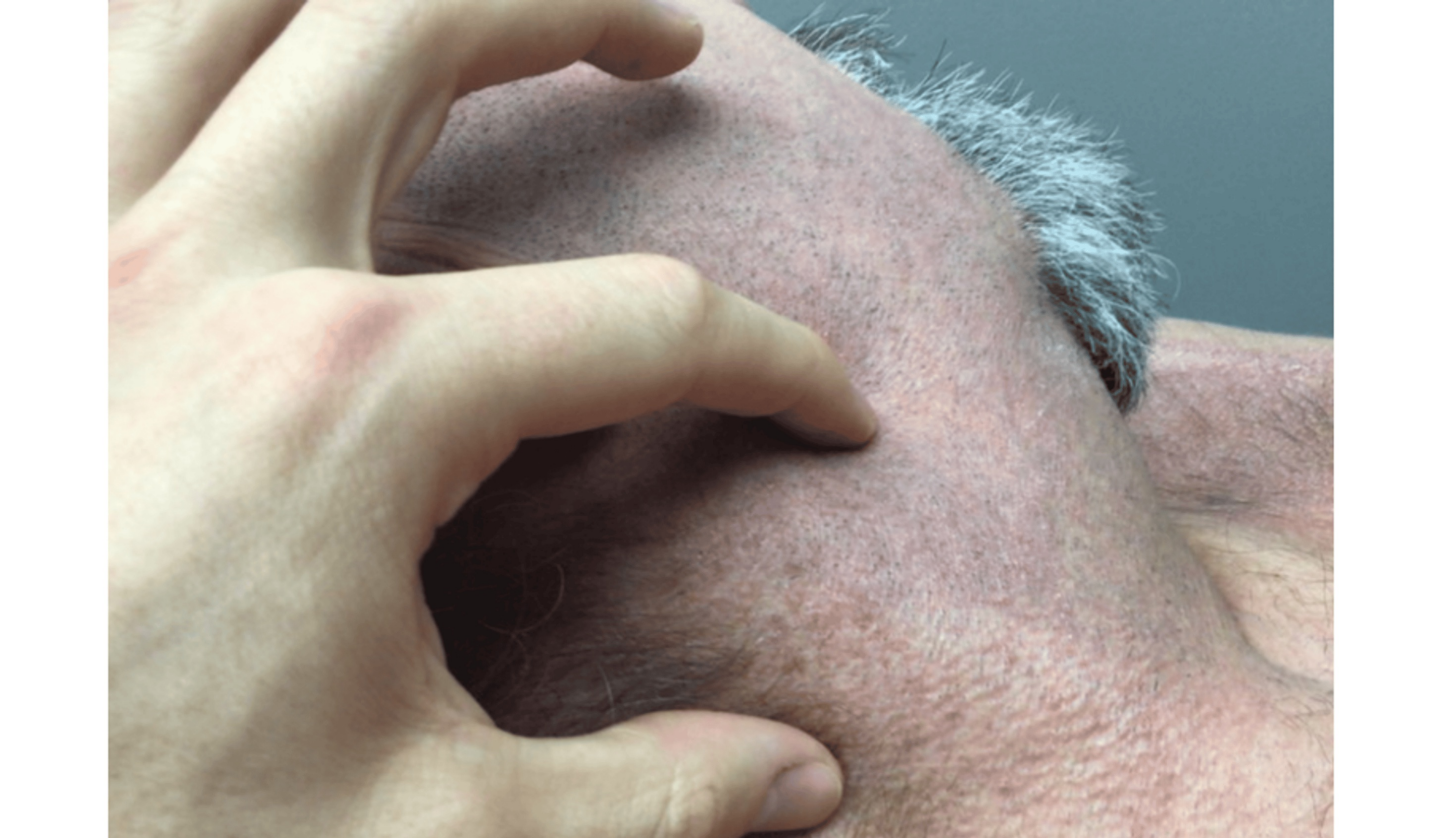
A 3 cm firm, painless nodule on the right lateral neck

**Figure 2:**
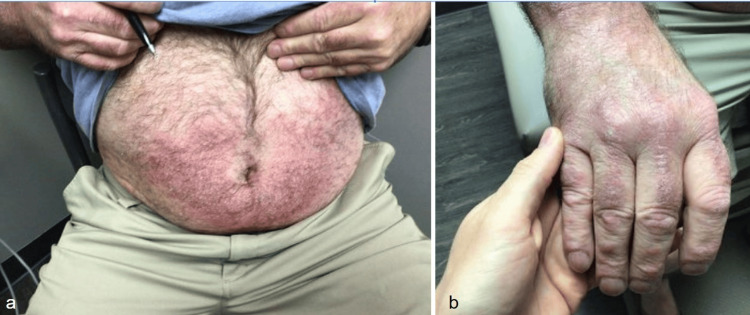
Psoriasiform plaques on the abdomen (a) and dorsal hand (b) where initial biopsies were taken

A 53-year-old Caucasian man with a past medical history of tobacco use disorder and a family history of psoriasis presented to the dermatology clinic with a one-year history of a progressive, non-pruritic psoriasiform eruption. The rash initially appeared on the dorsal hands and gradually spread to the dorsal forearms, trunk, and face. He denied a history of similar lesions, photosensitivity, or joint pain and had not seen a physician in many years. Physical examination revealed a 3 cm indurated, painless nodule on the right lateral neck, suspicious for an enlarged lymph node (Figure 1), in addition to the aforementioned rash. No other lymphadenopathy was detected on examination. A clinical diagnosis of psoriasis was made, and a confirmatory punch biopsy of the abdomen and shave biopsy of the dorsal digit were taken (Figure 2) to rule out a differential diagnosis that included psoriasis, dermatomyositis, pityriasis rubra pilaris, and mycosis fungoides. The patient was prescribed topical steroids with the intent to start a biologic medication pending bloodwork and biopsy results. The patient was also strongly encouraged to follow up with his primary care for further evaluation of the neck mass due to concern for malignancy. Histopathologic analysis revealed psoriasiform spongiotic dermatitis, a finding that excluded several conditions in the differential but was ultimately non-diagnostic.

**Figure 3:**
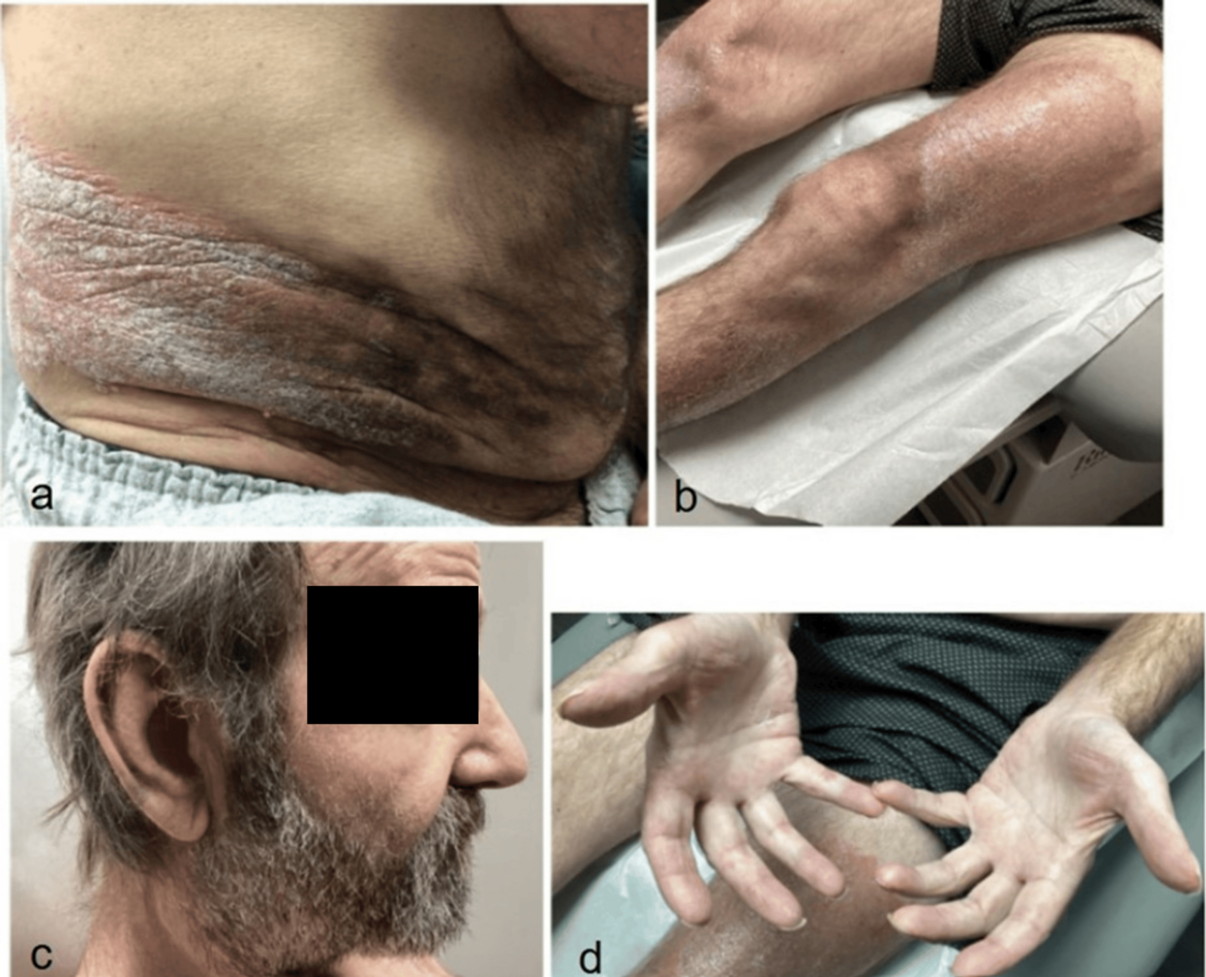
Psoriasiform plaques on the right lateral abdomen (a) and legs (b), as well as scaly, erythematous plaques on the right ear and nose (c) and erythematous plaques on the dorsal fingertips (d)

The patient returned to the clinic one year later with a markedly worsened clinical presentation. During the intervening period, he had been diagnosed with oral SCC with metastasis to the head and neck and was scheduled to begin chemotherapy. He now reported unintentional weight loss, night sweats, and what he described as skin thickening with decreased range of motion in the extremities, trunk, and digits. He also described new-onset joint pain, paresthesias of the hands and fingers, and nail clubbing (Figure 3). Lymph node exam again demonstrated an indurated subcutaneous nodule on the right lateral neck.

Due to the progression of the patient's condition, four additional punch biopsies were obtained from the arm, thigh, upper chest, and abdomen. Histopathologic evaluation of all specimens now revealed chronic interface dermatitis with dermal mucin deposition. Bloodwork revealed an unremarkable complete blood count (CBC) and comprehensive metabolic panel (CMP), elevated total and low-density lipoprotein (LDL) cholesterol and triglycerides, and low high-density lipoprotein (HDL) cholesterol. It also showed a negative QuantiFERON Gold and antinuclear antibody (ANA) as well as low vitamin D. Given that these findings can be characteristic of connective tissue diseases, the patient was referred to rheumatology for further evaluation and laboratory workup. Unfortunately, he was subsequently lost to follow-up, and no additional diagnostic information could be obtained.

Based on the clinical presentation and histopathologic findings, the primary differential diagnoses considered were systemic lupus erythematosus, dermatomyositis, and Bazex syndrome. The constellation of a Caucasian man in his 50s with metastatic oral SCC and a preceding diffuse psoriasiform eruption involving typical sites for Bazex-albeit an atypical progression-along with non-specific histopathology that is not consistent with psoriasis, points toward Bazex syndrome (acrokeratosis paraneoplastica) as the most likely diagnosis. While dermatomyositis was considered, it is more frequently associated with breast, ovarian, lung, and hematologic malignancies in North America and less commonly with oral SCC [10,11]. Systemic lupus erythematosus was considered likely given the lack of a well-established association with oral SCC as a paraneoplastic trigger.

This case illustrates an atypical presentation of Bazex syndrome and emphasizes the diagnostic value of comprehensive physical examinations-particularly lymph node assessment. The recognition of the lateral neck mass and the recommendation for primary care follow-up may have played a key role in prompting the eventual diagnosis of malignancy. This case also reinforces the importance of maintaining a broad differential in patients with persistent psoriasiform eruptions, particularly when the presentation deviates from classic psoriasis and is accompanied by systemic symptoms.

## Discussion

Bazex syndrome, also known as acrokeratosis paraneoplastica, is a rare paraneoplastic dermatosis strongly associated with SCC of the upper aerodigestive tract [12]. Other neoplastic associations include SCC and adenocarcinoma of the lung as well as gastric and colonic adenocarcinoma [9]. It primarily affects Caucasian men between the ages of 40 and 80, as reflected in our case [12]. Clinically, it presents as psoriasiform plaques involving acral regions-most commonly the hands, feet, and ears-and often precedes the diagnosis of an underlying malignancy, typically resolving with successful cancer treatment [9]. Although the exact pathogenesis of Bazex syndrome remains unclear, several mechanisms have been proposed. These include tumor-secreted factors affecting epithelial cells, an immune shift toward Th2 cytokine release increasing epidermal growth factor receptor (EGFR) expression in keratinocytes, direct tumor secretion of growth factors stimulating epithelial hyperproliferation, and tumor-derived antigens triggering a cross-reactive antibody response against keratinocytes [5,13].

The disease is classically described as progressing through three stages: initial involvement of the fingernails, nose, and ears; subsequent spread to the palms and soles; and eventual extension to the trunk [12]. Notably, cutaneous manifestations may precede cancer detection by several months to a year, as was the case in our patient. Interestingly, our case deviated from the typical progression. Although the palmar hands and ears were eventually involved, the eruption began on the dorsal hands, trunk, and legs. This presentation highlights a less common sequence of lesion development in Bazex syndrome. Importantly, while the patient's cutaneous findings preceded the diagnosis of malignancy, an indurated neck nodule-later confirmed as metastatic SCC-was already present at his initial dermatologic evaluation, highlighting the importance of a thorough physical examination. Generally, additional features that may indicate underlying malignancy include pruritus, ichthyosis, Sister Mary Joseph nodule (palpable nodule bulging into the umbilicus), clubbing, sign of Leser-Trélat (diffuse eruption of seborrheic keratoses), and Virchow's nodes (enlarged lymph node in the left supraclavicular fossa) [12].

Our case resembles several key features of Bazex syndrome described in the literature, including the characteristic psoriasiform eruption affecting acral and facial sites and a strong association with upper aerodigestive tract malignancies such as oral SCC [9]. The patient's progressive cutaneous findings and systemic symptoms, including new-onset joint pain and nail clubbing, are similar to those described in prior case reports [4,13].

Diagnosis requires a biopsy to exclude psoriasis alongside a thorough clinical evaluation and relevant laboratory tests. Histopathology is varied but can include spongiosis and commonly reveals an interface dermatitis, which may resemble other lichenoid dermatoses such as lichen planus, lupus erythematosus, or a lichenoid drug reaction [9,12]. This was consistent with the spongiosis observed in our initial shave biopsy and the interface dermatitis found in our patient's subsequent punch biopsies. Primary treatment focuses on managing the underlying malignancy, though lesion reduction has been reported with PUVA, topical retinoids, vitamin D3, salicylic acid, and topical steroids [12,14]. The lesions are unlikely to resolve without treatment of the underlying malignancy [14]. If Bazex syndrome is suspected without a known diagnosis of malignancy, a thorough workup is indicated. This includes an ear, nose, and throat referral for otolaryngologic examination and oncology for optimal screening, as well as chest radiography, erythrocyte sedimentation rate, CBC and metabolic profile, tumor markers, and fecal occult blood testing [9].

The clinical significance of this case extends beyond its atypical morphologic progression. It highlights the pivotal role of the dermatologist in the detection of occult malignancy through comprehensive physical examination. The identification of cervical lymphadenopathy at the initial dermatologic visit ultimately contributed to the patient's cancer diagnosis. This is consistent with the broader literature demonstrating that in the majority of Bazex syndrome cases, cutaneous findings precede the diagnosis of the underlying malignancy, providing a valuable opportunity for early intervention [5].

When a psoriasiform eruption is refractory to standard therapies, fails to conform to a classic psoriasis phenotype, or is accompanied by systemic symptoms such as weight loss, night sweats, or nail clubbing, clinicians should maintain a high index of suspicion for paraneoplastic etiologies [2,4]. In high-risk populations-particularly middle-aged Caucasian men with a history of tobacco or alcohol use-Bazex syndrome should be included in the differential diagnosis, and an appropriate malignancy workup should be initiated promptly [5,9].

## Conclusions

In conclusion, we report an atypical presentation of Bazex syndrome with a non-classical pattern of lesion progression. This case underscores the importance of considering paraneoplastic dermatoses in the differential diagnosis of persistent, unexplained psoriasiform eruptions, particularly in high-risk populations.

Ultimately, early recognition of paraneoplastic dermatoses such as Bazex syndrome has the potential to facilitate timely cancer diagnosis and treatment, theoretically improving patient outcomes. Increased awareness of atypical presentations, as illustrated by this case, is essential to reducing diagnostic delays and ensuring that patients receive the oncologic care they need.
